# Nettle Tea Inhibits Growth of Acute Myeloid Leukemia Cells In Vitro by Promoting Apoptosis

**DOI:** 10.3390/nu12092629

**Published:** 2020-08-28

**Authors:** Mohammad Hassan Hodroj, Nour al Hoda Al Bast, Robin I. Taleb, Jamilah Borjac, Sandra Rizk

**Affiliations:** 1 Department of Natural Sciences, Lebanese American University, Beirut 1102-2801, Lebanon; mohammadhassan.hodroj@lau.edu (M.H.H.); n.bast93@gmail.com (N.a.H.A.B.); robin.taleb@lau.edu.lb (R.I.T.); 2 Department of Biological Sciences, Faculty of Science, Beirut Arab University, Debbieh 1107-2809, Lebanon; j.borjac@bau.edu.lb

**Keywords:** acute myeloid leukemia, apoptosis, medicinal herbs, nettle tea, cancer, *Urtica dioica*

## Abstract

*Urtica dioica* (UD), commonly known as “stinging nettle”, is a herbaceous flowering plant that is a widely used agent in traditional medicine worldwide. Several formulations of UD leaf extract have been reported to exhibit anti-inflammatory and antioxidant properties, with anticancer potential. The current study investigated the possible anticancer properties of nettle tea, prepared from *Urtica dioica* leaves, on acute myeloid leukemia (AML) cell lines, and deciphered the underlying molecular mechanisms. Treatment of AML cell lines (U-937 and KG-1) with UD aqueous leaf extract resulted in a dose- and time-dependent inhibition of proliferation, an increase in apoptotic hallmarks such as phosphatidylserine flipping to the outer membrane leaflet, and DNA fragmentation as revealed by cell-death ELISA and cell-cycle analysis assays. Apoptosis induction in U937 cells involves alterations in the expression of Bax and Bcl-2 upon exposure to nettle tea. Furthermore, the chemical composition of UD aqueous extract indicated the presence of multiple chemical agents, such as flavonoids and phenolics, mainly patuletin, m/p-hydroxybenzoic acid, and caffeic acid, among others, to which the pro-apoptotic and anti-tumor effects may be attributed.

## 1. Introduction

Acute myeloid leukemia (AML) is considered the most common type of acute leukemia among adults, accounting for around 80% of leukemia cases in this population [1]. Its incidence increases with age, along with an increase in severity and a decrease in survival rates [2]. Currently, combinations of chemotherapeutic drugs constitute the backbone of AML treatment, with a wide spectrum of different side effects, starting with anemia, hair loss, and diarrhea, in addition to nervous and fertility problems later [3]. Over the years, the serious adverse effects of chemotherapy treatment have stimulated researchers to divert their focus towards natural compounds that may be effective in treating various malignancies, including leukemia, with lower toxicity [4,5,6,7].

*Urtica dioica* (UD), a member of the Urticaceae family, is a herbaceous, perennial, and annual-flowering plant that grows in wet, rich soils with a height ranging between 0.5–1 m. It has fleshy, drooping, serrated, and roughly heart-shaped leaves, and the leaves and stem are covered with small, erect stinging hairs, for which the plant is known as the “stinging nettle’’ [8]. These stinging hairs contain high concentrations of formic acid and histamines that induce a burning sensation upon friction, an attribute that led to the Latin-derived name Urtica, which means to sting and burn [9]. Geographically, the wide distribution of UD in Asia, Europe, North America, and North Africa has contributed to its popularity in herbal medicine throughout history [10,11].

Over many centuries, UD has been a famous plant in traditional medicine, where the whole plant, leaves, stem, and roots are used to treat anemia, burns, kidney stones, and rashes [12]. The nettle extract has been shown to be involved in multiple biological and biochemical activities as part of phytotherapy, which explains its potential in treating different disorders that affect the skin, gastrointestinal tract, joints, genito-urinary tract, and benign prostatic hyperplasia [13,14]. Its exact chemical composition is still not clear, especially given that the proportion of the constituents varies from one part of the plant to another [15].

UD is rich in essential amino acids; several minerals such as calcium, iron, magnesium, and phosphorus; and vitamins like C, B, and K [13]. It was widely used by Western herbalists in tea, juice, green vegetable, and dried products for blood nourishment, as well as a remedy for allergic rhinitis [16]. Moreover, it contains flavonoids and aromatic compounds that exhibit antioxidant activity, besides the anti-inflammatory activity of its polysaccharides [8,17]. Despite all these studied effects of UD extracts, data showing clinical evidence for its anti-cancer effect are scarce and limited to a few in vitro studies on breast, prostate, and, recently, lung cancer cell lines, in addition to some animal models, that involved different formulations of UD leaf extract [17,18]. For instance, a study conducted by Mohammadi et al. showed that the dichloromethane extract of UD leaves inhibited the growth and proliferation of human prostate cancer cells (PC3), with an IC_50_ concentration of 15.54 µg/mL after 48 h of treatment [19]. In addition, recent studies have focused on its synergistic effect on cancer cell lines when combined with standard chemotherapy [20].

The current study aimed to investigate the anti-proliferative activity of nettle tea, which is the aqueous *Urtica dioica* leaf extract, on AML cell lines and to decipher the underlying molecular pathways involved in U937 cells.

## 2. Materials and Methods

### 2.1. Preparation of UD Aqueous Extract

The leaves of *Urtica dioica* were collected from Bawarij, Lebanon (33°49′0” N 35°49′0” E 1249 m above sea level) in September 2018, where they were identified based on characteristics described by Weigend [21], and then identified by an expert on Lebanese flora, Dr. Nisrine Machaka-Houri [22]. A voucher specimen was deposited in the Beirut Arab University Herbarium (ID- RCED2019-363).

Nettle tea was prepared from fresh leaves (20 g) which were washed, cut into small pieces, and kept in boiling distilled water (100 mL) for 30 min with mild shaking, producing a clear liquid bearing a dark green color. This aqueous extract (20% w/w) was centrifuged and distributed into aliquots, and then stored at −20 °C. On the day of treatment, an aliquot was thawed, filtered through a 20 μm Millipore, and applied by adding different volumes from the stock to cells in culture.

### 2.2. Cell Culture

The AML cell lines U937 and KG-1 were obtained from the American Type Culture Collection. The normal human B-lymphocyte (BLH) cell line (GM03299) was purchased from Coriell Cell Repository. Cells were cultured in Roswell Park Memorial Institute medium (RPMI-1640 Sigma-Aldrich) supplemented with 10% Fetal Bovine Serum (FBS GibcoTM) and antibiotics (100 U/mL penicillin and 100 µg/mL streptomycin from Pen-Strep Lonza), as previously described. They were split every 3 days at a ratio of 1:2. Cell viability was continuously checked using the trypan blue exclusion method [23].

### 2.3. Cytotoxicity Assay

U937 and BLH cells were seeded in triplicate in 96 well plates at a density of 1 × 10^4^ cells/well for 4 h before treatment. For dose- and time-dependent viability assays, different volumes of UD extract ranging from 2–12 μL were added over 24, 48, and 72 h, yielding final extract concentrations of 0.4–2.4%. Controls were treated with medium only. The chemotherapeutic drug topotecan (100 nM) was used as a positive control [24]. The cell viability reagent (Vita-Orange, Biotool) was then added according to the manufacturer’s guidelines. Spectrophotometry using Varioskan Flash (Thermo SCIENTIFIC) was adopted to measure the absorbance at 450 nm in order to detect metabolically active cells and calculate the percentage proliferation of control cells.

### 2.4. Cell-Cycle Analysis

Propodium iodide (PI) staining was used to assess the effect of different concentrations of UD aqueous extract on the cell-cycle profiles of U937 cells at 48 and 72 h post-treatment. Six well plates were seeded with cells at a density of 1 × 10^5^ cells/mL for 4 h before treating with UD extract to final concentrations of 0.4, 0.8, and 2.4% (concentrations above and below the IC_50_ values obtained in the cytotoxicity assay). Untreated cells were used as controls. Cells were fixed with ethanol and then stained with PI after treatment for 48 and 72 h in order to assess the cells’ DNA content using an Accuri C6 flow cytometer [25]. Cells were distributed into distinct cell-cycle phases according to their DNA content: cells were <2n in sub-G0/G1 (pre-G0), 2n in G0/G1, >2n but <4n in S phase, and G2/M-phase cells were 4n. An increase in the percentage of cells in the pre-G0 phase indicated an increase in cell fragmentation or cell death.

### 2.5. Apoptosis Detection

Six well plates were used to seed U937 cells at a density of 1 × 10^5^ cells/well for 4 h before treatment with the following concentrations of UD extract: 0.4, 0.8, 1.6, and 2.4%. Untreated cells were used as controls. Cells were collected and centrifuged after 48 and 72 h of treatment, and the pellets were stained with both annexin V and PI (annexin V–fluorescein isothiocyanate (FITC) Apoptosis Detection Kit, Abcam, Cambridge, UK) and immediately analyzed using the Accuri C6 flow cytometer, as previously described [26,27]. Upon apoptosis induction, phosphatidyl serine is translocated to the outer leaflet of the cell membrane, and living cells exclude PI from the cytoplasm. Therefore, living cells will stain negative for both FITC–annexin V and PI, whereas those that stain positive for both can be classified as apoptotic dead cells [28].

### 2.6. Assessment of DNA Fragmentation Using Cell-Death ELISA

A 24 well plate was used to seed cells at a density of 1 × 10^5^ cells/well and incubated for 4 h, before treatment with 0.4, 0.8, 1.6, or 2.4% of aqueous UD extract for 48 and 72 h. Cells were extracted and lysed using incubation buffer provided with the cell-death ELISA kit (Roche), before isolation of fragmented cytosolic DNA as previously described [29]. Briefly, extracted DNA was incubated in microplate wells pre-coated with anti-histone antibodies. The wells were washed prior to adding anti-DNA antibodies linked to an enzyme, and then washed again prior to the addition of the provided colorimetric substrate, ABTS. The absorbance was recorded at 405 nm using ELx808 BioTek spectrophotometer plate reader in order to calculate the DNA fragmentation enrichment factor.

### 2.7. Western Blot Analysis

Petri dishes were used to seed the cells at a density of 5 × 10^5^ cells/mL. Total proteins were extracted using the Q-proteome Mammalian Protein kit following 48 and 72 h of treatment with 0.4–2.4% of UD aqueous extract. Lowry assays were used for protein quantification. Western blot analysis was done to measure the protein expression of Bax and Bcl2. Beta-actin was used as a loading control. Proteins were separated on 10% sodium dodecyl sulfate–polyacrylamide gels, and then transferred to polyvinylidene difluoride membranes at 0.25 mA for 75 min [30]. After blocking with 5% milk and 0.05% Tween 20 for 1 h, the membranes were incubated with the antibodies using anti-β-actin (Sc-47778), anti-Bax (Sc-7480), and anti-Bcl2 (Sc-783) overnight at 4 °C (Santa Cruz Biotechnology, Dallas, TX, USA) at a concentration of 1:2500 for anti-β-actin and 1:500 for the others. The membranes were then washed for 1 h using 1 × PBS with 0.5% Tween 20, before incubation with the secondary antibody (Promega, Madison, WI, USA) at a concentration of 1:2500 for 1 h at room temperature. After washing, the development of the membranes was done using Western blotting chemiluminescent-reagent-enhanced chemiluminescence (GE Healthcare, Chicago, IL, USA), and a ChemiDoc XRS+ machine (BioRad, Hercules, CA, USA) was used to take images that were quantified and analyzed using Image J software [31].

### 2.8. Extraction and Chemical Analysis

#### 2.8.1. Extraction and Ultra-Performance Liquid Chromatography (UPLC) Analysis of Polyphenols, Terpenes, Sesquiterpenes, Fatty Acids, and Flavonoids

The aqueous UD extract was filtered with 0.25 µm Millipore SPE cartridges and diluted 1:10 with liquid chromatography mass spectrometer (LCMS)-grade water. The resultant crude solution was injected into a UPLC-Photodiode Array (Thermo Scientific, Waltham, MA) using a C18-Hypersil Gold reverse-phase column to acquire a fingerprint 3D chromatogram. Gradient elution was performed with water/acetonitrile at a constant flow rate of 0.285 mL/min and an injection volume of 5 µL. Separation was carried out over 30 min under the following conditions: 0 min, 95% water; 2.192 min, 75% water; 4.38 min, 75% water; 6.577 min, 63% water; 9.5 min, 63% water; 13.15 min, 46% water; 16.1 min, 46% water; 19.0 min, 5% water; 21.2 min, 5% water; 21.5 min, 95% water; 30 min, 95% water.

#### 2.8.2. LC-TSQ-Endura-MS/MS Analysis

Screening for polyphenols, terpenes, sesquiterpenes, fatty acids, and flavonoids was carried out via direct injection (1 ppm, 2 µL) into a UPLC-TSQ-Endura Triple Quadruple mass spectrometer (Thermo Scientific, Waltham, MA) equipped with an ESI source operating in both positive- and negative-ion modes. In positive-ionization mode, the mobile phase used was 10% methanol:water in formic acid at a flow rate of 250 µL/min, while in negative-ionization mode, the same mobile phase was used but without formic acid. Mass spectrometer parameters were as follows: spray voltage, ±3500 V; sheath gas, 25; auxiliary gas, 10; sweep gas, 0; ion transfer tube temperature, 329 °C; and vaporizer temperature, 296 °C. The UD aqueous extract was screened for 50 polyphenols, terpenes, sesquiterpenes, fatty acids, and flavonoids (Table 1). Screening and detection of these compounds was carried out using MRM experiments for which parent and product ions and the collision energies are reported in Table 2.

### 2.9. Statistical Analysis

All experiments were performed in triplicate and each experiment was repeated three independent times. The results were reported as mean ± SEM. The analysis was done using two-way analysis of variance (ANOVA) followed by Tukey’s multiple comparison test. The level of significance upon comparing control versus treatment was set at *p* < 0.05.

## 3. Results

### 3.1. Cytotoxic Effect of UD Extract on AML and Normal B-Lymphocyte Cells

**Figure 1:**
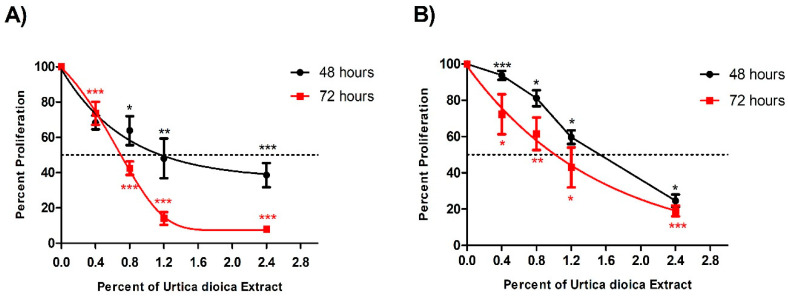
Effect of varying UD extract concentrations on the viability of U937 and KG-1 cells after 48 and 72 h. (**A**) U937 cells showed a significant decrease in proliferation, with an IC_50_ at 1.1% of UD extract after 48 h and at 0.7% after 72 h. (**B**) KG-1 cells showed a significant decrease in proliferation, with an IC_50_ at 1.5% of UD extract after 48 h and at 1.0% after 72 h. The figure represents the results from three independent experiments and the error bars represent SEM. *, **, and *** indicate *p* < 0.05, *p* < 0.001, and *p* < 0.0001, respectively, compared to the control group.

UD aqueous extract was used on U937 and KG-1 cells at different percentages ranging from 0.4% to 2.4%, and it induced a significant anti-proliferative effect in a dose- and time-dependent manner in both cell lines, with a half-maximal inhibitory concentration (IC_50_) seen at 1.1% and 1.5% after 48 h, and at 0.7% and 1.0% after 72 h treatment in U937 and KG-1 cells, respectively. The significant reduction in the proliferation observed at 2.4% UD was more prominent after 72 h of treatment, reaching 81% compared to 68.5% after 48 h (Figure 1A). Similarly, the significant reduction in proliferation of KG-1 cells observed at 2.4% UD was more prominent after 72 h, with 81% compared to 75.4% after 48 h (Figure 1B). The proliferation of cells treated with topotecan (100 nM) for 48 h as a positive control was reduced to 19% (±4) and 22% (±3) in U937 and KG-1 cells, respectively. The UD effect was more potent on U937 cells, with lower IC_50_ concentrations in both arms of treatment. For this reason, the U937 cell line was used for all the remaining experiments.

**Figure 2:**
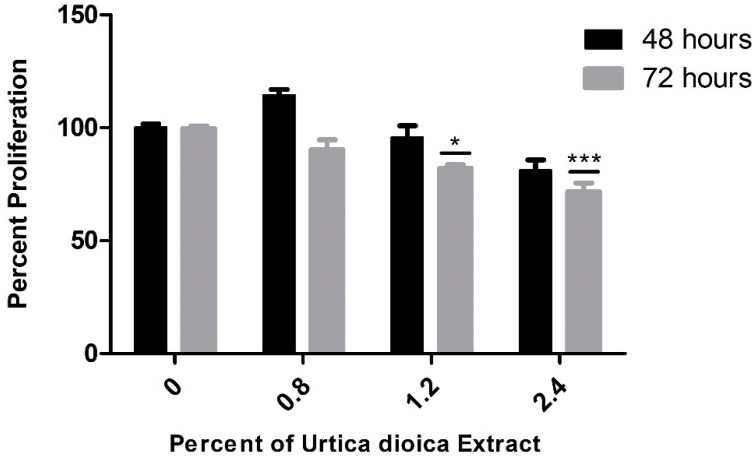
Effect of varying UD extract concentrations on the viability of normal B-lymphocyte cells after 48 and 72 h. UD extract exerted no major effect on the B lymphocytes after 48 h, with a minimal reduction effect after 72 h at the highest concentrations. The graph represents the means from three independent experiments +/− SEM. * and *** indicate *p* < 0.05 and *p* < 0.0001, respectively, compared to the control group.

Interestingly, when applied on the normal human B-lymphocyte cell line, aqueous UD extract did not exert major effects on the viability of these cells after 48 h, with minimal reduction after 72 h mainly detected at the highest concentration, implying its selectivity and safe use at the studied concentrations (Figure 2).

### 3.2. Effect of UD Extract on Cell-Cycle Progression in U937 Cells

**Figure 3:**
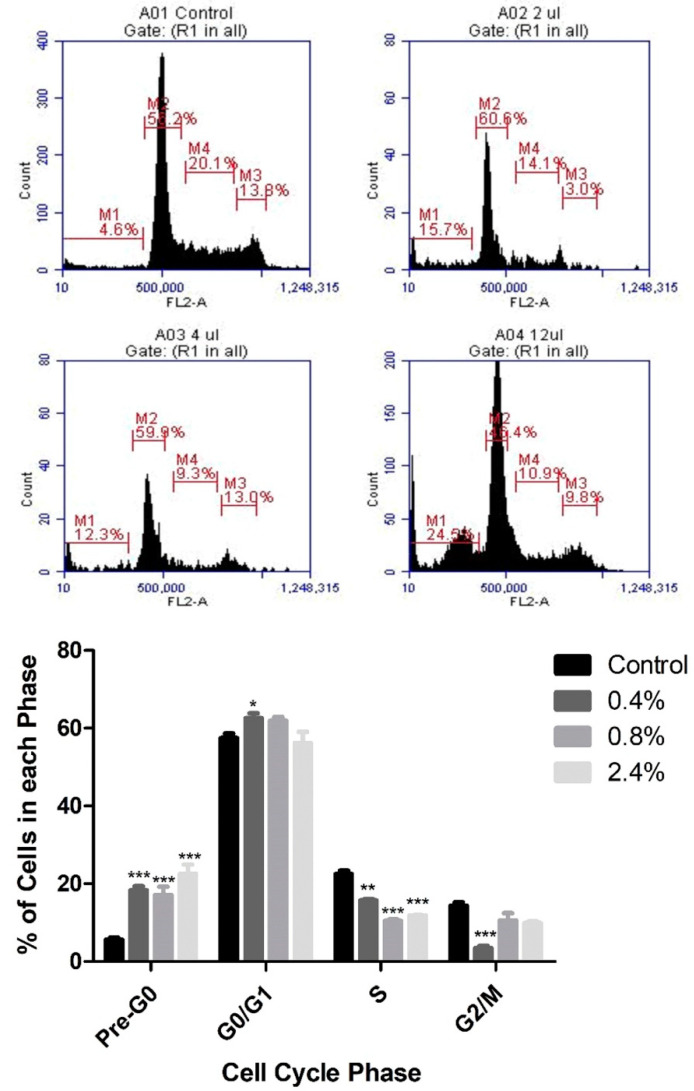
Dose-dependent effect of UD extract on cell-cycle distribution of U937 cells after 48 h of treatment. Cells are distributed into the different phases of the cell cycle according to their DNA content: pre-G0 cells are <2n (M1), G0/G1 cells are 2n (M2), S cells are >2n but <4n (M4), and G2/M-phase cells are 4n (M3). The histogram represents the means from three independent experiments +/− SEM. *, **, and *** indicate *p* < 0.05, *p* < 0.001, and *p* < 0.0001, respectively, compared to the control group.

**Figure 4:**
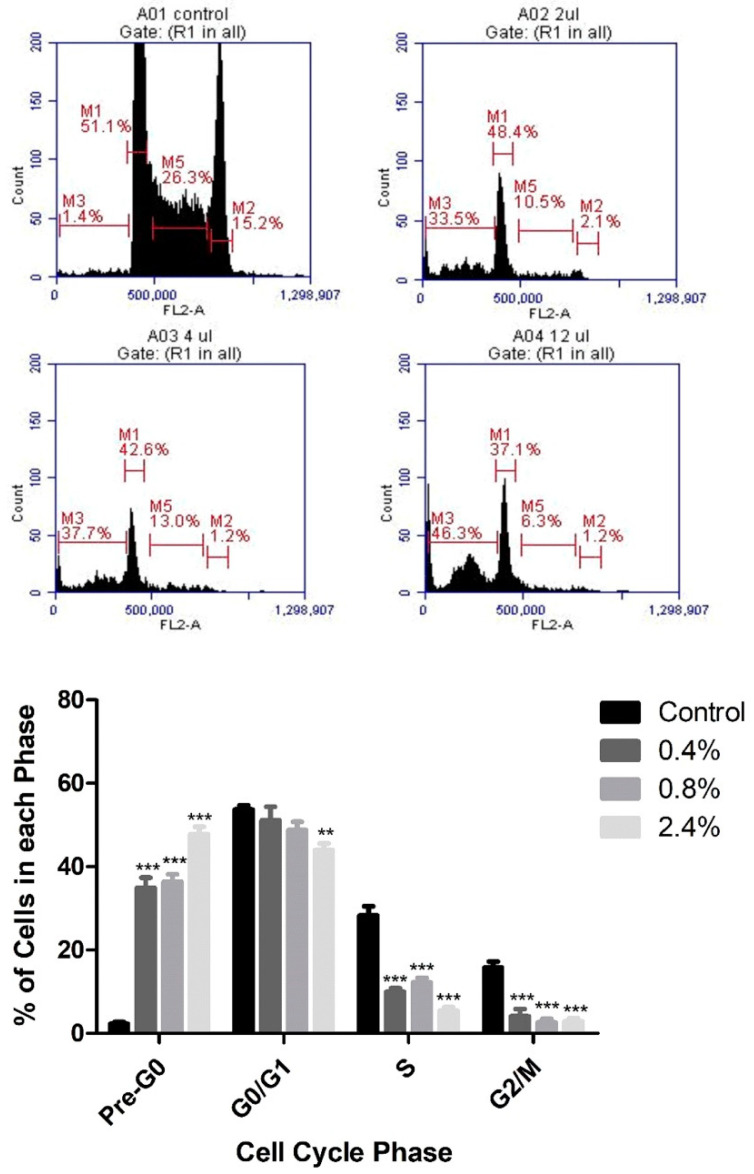
Dose-dependent effect of UD extract on cell-cycle distribution of U937 cells after 72 h treatment. Cells are distributed into the different phases of the cell cycle according to their DNA content: pre-G0 cells are <2n (M3), G0/G1 cells are 2n (M1), S cells are >2n but <4n (M5), and G2/M-phase cells are 4n (M2). The histogram represents the means from three independent experiments +/− SEM. ** and *** indicate *p* < 0.001, and *p* < 0.0001, respectively, compared to the control group.

Based on the cytotoxicity results, PI staining and flow cytometry were used to assess the effects of three concentrations of UD extract (0.4%, 0.8%, and 2.4%) on cell-cycle arrest of U937 cells. UD extract caused a dose-dependent increase in the percentage of cells in pre-G0 phase, reaching 22.5% at the highest concentration of UD compared to the untreated cells after 48 h (Figure 3). On the other hand, the increase in the percentage of cells in pre-G0 phase was more significant after 72 h of treatment, reaching 47.79% at the highest concentration of UD extract (Figure 4).

### 3.3. Effect of UD Extract on Apoptosis Induction in U937 Cells

**Figure 5:**
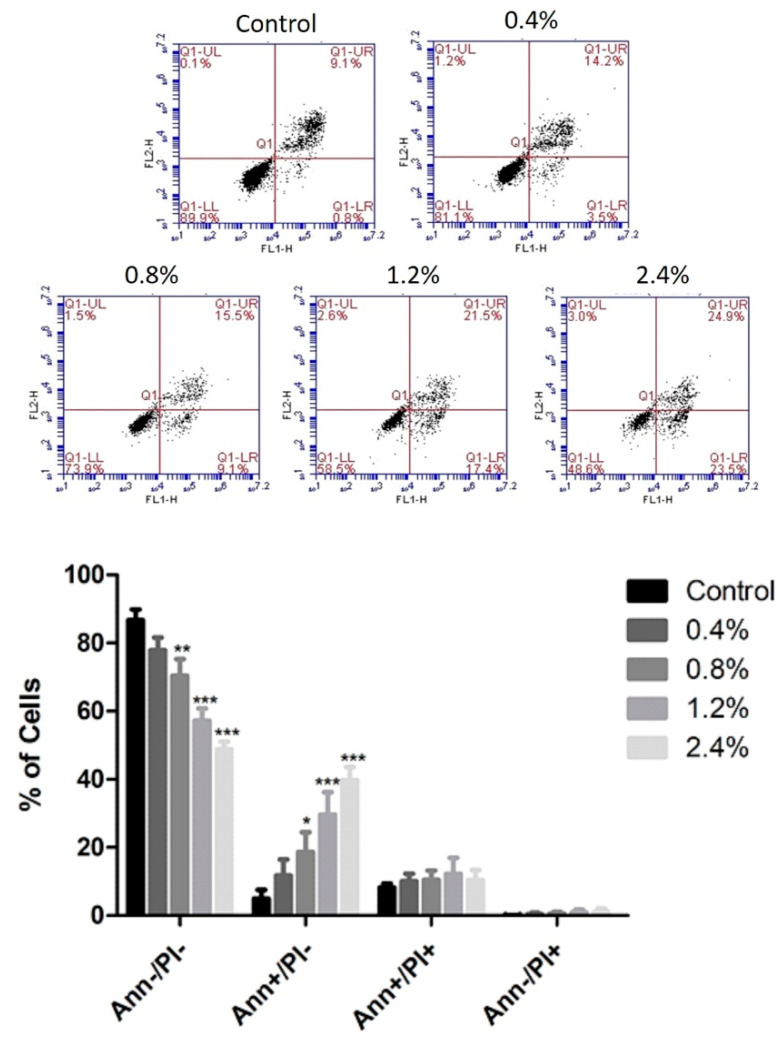
Effect of UD extract on apoptosis after 48 h. U937 cells were treated with different concentrations of UD extract at 0.4, 0.8, 1.2, and 2.4%. The cells were stained with annexin V/PI after 48 h, and then analyzed using flow cytometry. The x-axis represents annexin V and the y-axis represents PI. Lower left quadrant represents Ann−/PI−cells, lower right quadrant represents Ann+/PI−cells, upper left quadrant represents Ann−/PI+ cells, and upper right quadrant represents Ann+/PI+ cells. The histogram represents the means from three independent experiments +/−SEM. *, **, and *** indicate *p* < 0.05, *p* < 0.001, and *p* < 0.0001, respectively, compared to the control group.

**Figure 6:**
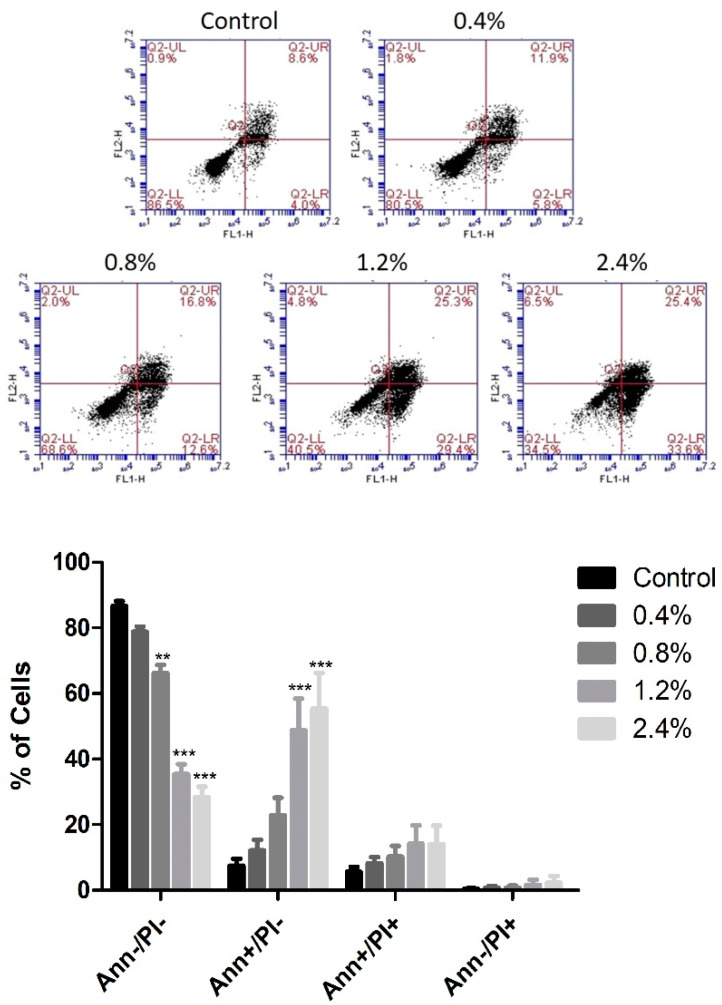
Effect of UD extract on apoptosis after 72 h. U937 cells were treated with different concentrations of UD extract at 0.4, 0.8, 1.2, and 2.4%. The cells were stained with annexin/PI after 72 h, and then analyzed using flow cytometry. The x-axis represents annexin V and the y-axis represents PI. Lower left quadrant represents Ann−/PI− cells, lower right quadrant represents Ann+/PI− cells, upper left quadrant represents Ann−/PI+ cells, and upper right quadrant represents Ann+/PI+ cells. The histogram represents the means from three independent experiments +/− SEM. ** and *** indicate *p* < 0.001 and *p* < 0.0001, respectively, compared to the control group.

Annexin V/PI staining was used to determine whether U937 cells died of apoptosis or necrosis upon the application of the several concentrations of aqueous UD extract ranging between 0.4% and 2.4%. UD treatment for 48 h induced a dose-dependent increase in the number of apoptotic cells from 13.16% to 49.9% compared to control cells (Figure 5). However, a more prominent apoptotic effect was seen upon 72 h of UD treatment, with an increase from 13.03% to 69.36% at the highest concentration of UD extract (Figure 6).

**Figure 7:**
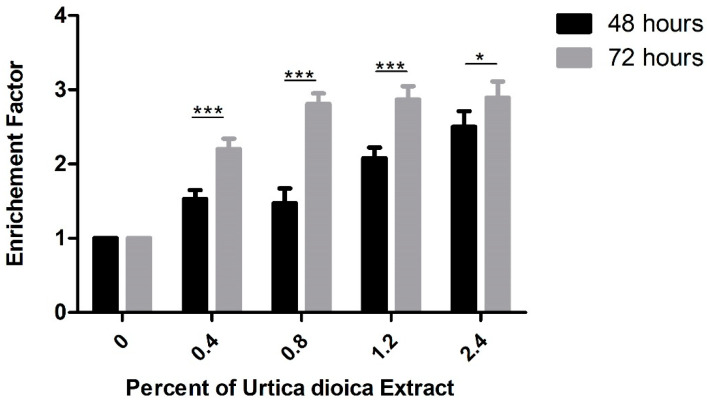
Cell-death ELISA showing the effect of UD extract on increasing DNA fragmentation in U937 treated with different concentrations of UD extract at 0.4, 0.8, 1.2, and 2.4% after 48 and 72 h. The graph represents the means from three independent experiments +/− SEM. * and *** indicate *p* < 0.05 and *p* < 0.0001, respectively, compared to the control group.

For further confirmation of the increase in apoptosis induced by aqueous UD extract, DNA fragmentation, which is considered a hallmark of apoptosis, was assessed using cell-death ELISA. A significant dose-dependent increase in the enrichment factor was detected in U937 cells after 48 and 72 h of treatment with UD extract. A significant 2.5-fold increase in the enrichment factor was observed in U937 cells at 2.4% UD after 48 h, compared to a more prominent significant increase of almost 3-fold at the same concentration after 72 h (Figure 7).

### 3.4. Effect of UD Extract on the Expression of Proteins Involved in Apoptosis Induction

**Figure 8:**
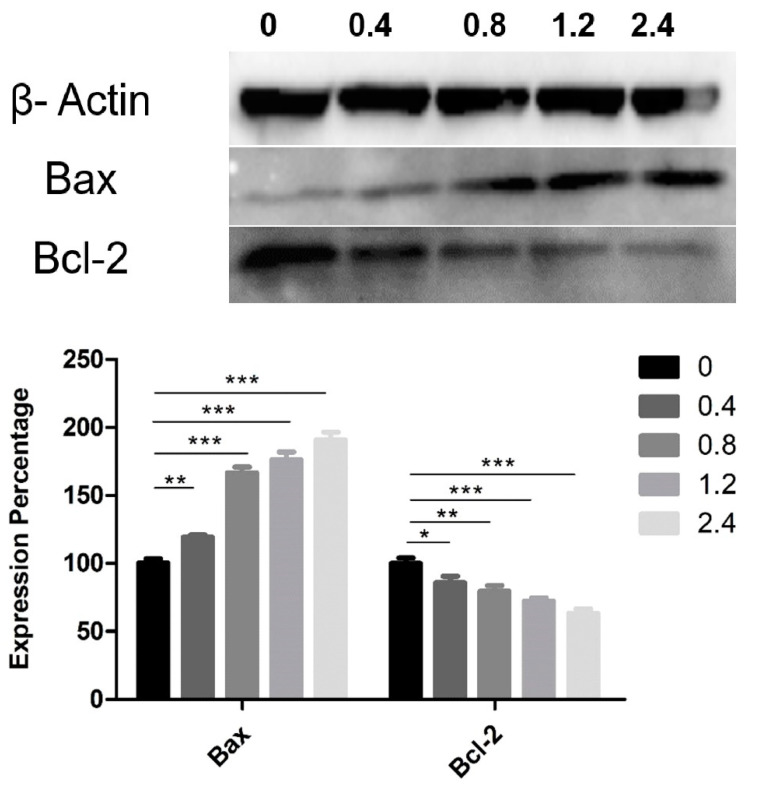
Effect of UD extract on the expression level of Bax and Bcl-2 after 48 h of treatment with different concentrations of UD extract at 0.4, 0.8, 1.2, and 2.4%. β-Actin was used as a loading protein. The graph represents the means from three independent experiments +/−SEM. *, **, and *** indicate *p* < 0.05, *p* < 0.001, and *p* < 0.0001, respectively, compared to the control group.

Western blotting was used to determine the expression of Bax and Bcl-2 proteins, which belong to a family of proteins involved in cell-death molecular pathways. Aqueous UD extract induced an upregulation in the expression level of the pro-apoptotic protein Bax, with a reduction in the expression of the anti-apoptotic protein Bcl-2 in U937 cells after 48 h as compared to the controls (Figure 8). UD extract induced a significant dose-dependent increase in the expression level of Bax, reaching 90.9% (*p* < 0.0001) at the highest concentration. In contrast, Bcl-2 showed a significant decrease in its expression level by 36.9% (*p* < 0.0001) at the same UD concentration after 48 h of treatment (Figure 8). The highest concentration of UD extract increased the Bax/Bcl-2 ratio 3-fold after 48 h of treatment.

### 3.5. Chemical Profile of the Aqueous Extract of UD Leaves

**Figure 9:**
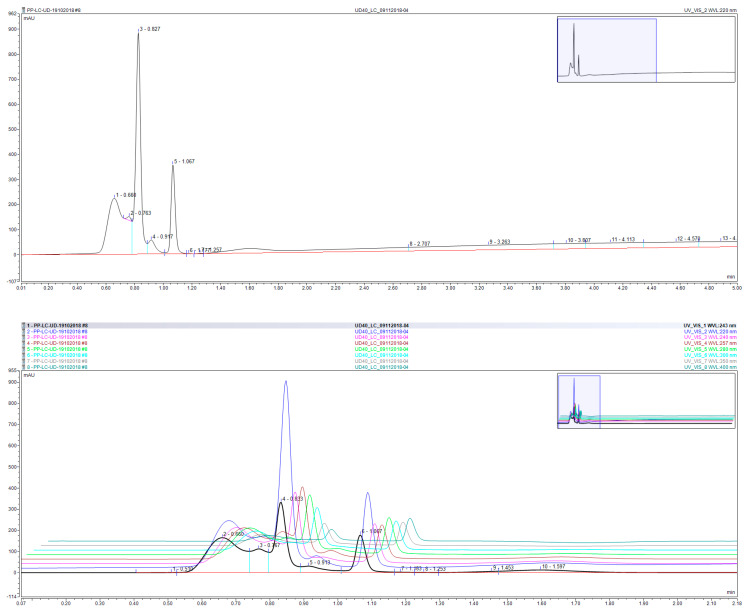
UPLC chromatogram (top) and 3D chromatogram (bottom) of UD leaf aqueous extract representing absorbance (y-axis) as a function of time (x-axis). The 3D chromatogram is reported at wavelengths 220 (blue), 240 (pink), 243 (black), 257 (brown), 280 (green), 300 (cyan), 350 (grey), and 400 (navy green) nm.

Chromatographic duplicate analysis (Figure 9) of the aqueous extract of UD leaves revealed the presence of three major components, with minimal column retention and elution within 1.2 min of injection. Surprisingly however, the chromatogram of the extract, albeit crude, did not show a significant number of peak, which may be attributed to the excellent eluting power of the column whereby polar analytes are quickly eluted out of the Hypersil Gold Column, and consequently several components may elute out at the same retention time. Moreover, the crude extract seemed to be saturated with three major components, rendering all other components as minor and presenting at concentrations below or close to the baseline.

A list of 50 common polyphenols, terpenes, sesquiterpenes, fatty acids, and flavonoids (Table 1) was formulated based on a literature review on the constituents of culinary and regional herbs [32,33,34]. These compounds were then analyzed via direct injection into a TSQ-Endura Triple Quadruple mass spectrometer in which detection and qualitative analysis were carried out based on MRM transitions reported by Vallverdú-Queralt et al. and using PubChem mass spectral data [34,35]. Out of the 50 polyphenols terpenes, sesquiterpenes, fatty acids, and flavonoids screened for, 22 were detected via direct injection into the MS, whereby detection was confirmed through a signal intensity in excess of e^1^ (Table 2). Among the detected compounds, the highest signal intensity was in the order of e^4^ and corresponded to patuletin, m/p-hydroxybenzoic acid, and caffeic acid. Signals in the order of e^3^ were observed for bisabolol oxide B, gallic acid, chlorogenic acid, homovanillic acid, and kaempferol-3-O-rutinoside.

## 4. Discussion

Even though herbal medicine is still lacking strong evidence for its role in treating cancer and improving patients’ outcomes, its use is prevalent among cancer patients worldwide. This use is supported by a large body of evidence from clinical studies reporting the benefits of several herbal medicines in reducing chemotherapy-induced toxicities [36,37]. However, the research fields of herbal medicine in cancer have now shifted from reducing toxicities to a new perspective that investigates the presence of potential anti-tumor and anti-proliferative effects of multiple herbal agents, especially those that are consumable and can be used as functional food or dietary supplements, such as *Urtica* species [38,39]. UD is a popular herbal medicine with various applications, and that has been being extensively studied for years for these effects in vitro and recently through in vivo models [38,40].

UD extract inhibited the proliferation of breast cancer cells in a BALB/c mouse model, leading to a significant decrease in the tumor mass in a dose-dependent pattern compared to the control group. In addition, the extract suppressed the growth of the tumor cells as measured using Ki-67 tests [41]. Moreover, UD dried leaf aqueous extract exerted a dose-dependent antioxidant and apoptotic effects on breast cancer cell line MCF-7 after 72 h of treatment, with an IC_50_ of 2mg/mL [17]. Similarly, the same extract exerted a cytotoxic and apoptotic effects on prostate carcinoma LNCap cell line after 24 h of treatment with an IC_50_ of 42 µg/mL [18]. The current study demonstrated a similar dose- and time-dependent anti-proliferative effect of UD fresh leaf aqueous extract (nettle tea) on the rapidly proliferating U937 AML cell line, with similar results in the KG-1 AML cell line.

Mohammadi et al. showed that UD dichloromethane extract induced cell-cycle arrest of the colorectal cancer cell line HCT at the G2/M phase [42]. On the other hand, our study illustrated a significant dose-dependent increase in the percentage of cells in the pre-G0 phase, also known as the sub-G1 phase, which was more prominent after 72 h at lower UD concentrations. This accumulation of cells in the sub-G1 population indicated an increase in the internucleosomal discontinuous DNA fragmentation, which is one of the hallmarks of apoptosis and is consistent with our presented results for the cell-death ELISA [43].

UD extract enhanced the cytotoxicity of cisplatin in non-small-cell lung cancer H460, H1299, A549, and H322 cell lines through the induction of endoplasmic-reticulum-stress-mediated apoptosis [44]. Furthermore, it activated apoptosis by targeting the ornithine decarboxylase and adenosine deaminase pathway in breast cancer cell lines MCF-7 and MDA-MB-231 [45]. The extract also exerted an apoptotic effect on the AML cell line KG-1 [46]. Similarly, our results using annexin V–PI staining and the cell-cycle-arrest assay indicated the apoptotic effect of UD extract in the AML cell line U937. In addition, injecting UD extract intraperitoneally into a mouse model with breast cancer was able to elicit intrinsic apoptotic pathway activation through increased expression of the pro-apoptotic caspase-3 and downregulation of the anti-apoptotic Bcl-2 protein [47]. UD-extract-induced intrinsic pathway activation was also shown in a study by Mohammadi et al., with an increase in the expression of both caspase-3 and caspase-9 and a decrease in Bcl-2 expression [20]. Moreover, UD extract induced apoptosis in breast cancer cells, mainly MCF-7 cells, through an increase in the expression levels of Bax, a well-known pro-apoptotic protein involved in the intrinsic pathway [48]. The data mentioned above support the results of the current study, where the aqueous UD extract induced apoptosis with a significant increase in the expression of Bax and a significant downregulation of Bcl-2.

D’Abrosca et al. reported that the effect of UD extract was specific for non-small-cell lung cancer cells without generating any toxic effects when applied on normal lung cells [44]. This was similar to the effect of UD extract demonstrated in the current study, where no toxicities were seen in normal human B lymphocytes after 48 and 72 h of treatment.

The results of the chemical profile of nettle tea, prepared from fresh UD leaves in the present study, may help to understand the chemical agents and components underlying its effects in inhibiting cancer cells’ proliferation and inducing apoptosis. The extract was found to contain a dominant amount of patuletin, a known O-methylated flavonol. This flavonol was shown to possess anti-proliferative and pro-apoptotic effects in human breast cancer cell line SK-BR-3 [49]. Moreover, patuletin and quercetagetin inhibited proliferation with an apoptotic activity in several tumor cell lines [50]. Another compound that was prominent in our extract was m/p-hydroxybenzoic acid, a phenol derivative of benzoic acid that is considered an important dietary phenolic agent. Its role in inhibiting the proliferation of cancer cell lines was found to occur through activating apoptotic pathways [51]. Caffeic acid, a component detected in high concentrations in our extract, has also been studied for its apoptotic effect on cancer cells: it induced apoptosis in human cervical cells via the mitochondrial pathway, which included a downregulation of Bcl2 and a release of cytochrome c [52]. Other compounds detected in smaller amounts included bisabolol oxide B, gallic acid, chlorogenic acid, and homovanillic acid, which are all associated with anticancer properties. Bisabolol oxide B extracted from *Matricaria chamomilla* showed promising pro-apoptotic effects in highly malignant human glioblastoma and pancreatic carcinoma cells [53]. Gallic acid is known to possess anticancer activity in many types of cancers, including prostate, lung, gastric, colon, breast, cervical, and lymphoblastic leukemia [54]. Moreover, chlorogenic acid, which has been reported to be safe in humans, triggered programmed cell death in human solid-tumor cell lines from colon cancer, glioma, hepatoma, and lung cancer [55]. Finally, homovanillic acid has also been reported to induce apoptosis through oxidative stress in myeloid leukemic cells both in vitro and in vivo, with no toxic effects on mice [56].

## 5. Conclusions

Nettle tea, derived from the aqueous extract of *Urtica dioica* fresh leaves, showed a dose- and time-dependent anti-proliferative effect when applied on AML cells in vitro. The extract induced apoptosis activation in U937 cells through the Bax/Bcl-2 pathway, with discontinuous DNA fragmentation as revealed by the cell-death ELISA and cell-cycle analysis assays. Furthermore, the chemical composition of UD extract indicated the presence of multiple chemical agents such as flavonoids, terpenes, sesquiterpenes, fatty acids, and phenolics, which may be responsible for the apoptotic and anti-tumor effect.

The effects of UD extract on cancer cells’ progression could offer new hope and perspectives in the use of herbal medicines and their natural extracts for the treatment of different cancer types, especially with herbs that are consumable as foods or dietary supplements such as UD, which has no toxic effects on normal cells. However, further studies and investigations should focus on determining the underlying molecular pathways and confirming the anti-leukemic effect of nettle tea in vivo.

## Figures and Tables

**Table 1:** List of polyphenols, terpenes, sesquiterpenes, fatty acids, and flavonoids screened for via LCMS/MS.

	Compound		Compound
1	alpha-Bisabolol	26	Neochlorogenic acid
2	Chamazulene	27	Protocatechuic acid
3	Methyl angelate	28	Caffeic acid 2-glucoside
4	Angelic acid	29	Homovanillic acid 1-glucoside
5	Isobutyl angelate	30	Caffeic acid 3-glucoside
6	alpha-Farnesene	31	p-Hydroxybenzoic acid
7	alpha-Pinene	32	Chlorogenic acid
8	Nobilin	33	4-O-beta-D-Glucosyl-4-coumaric acid
9	3-Epinobilin	34	m-Hydroxybenzoic acid
10	Bisabolol oxide A	35	Cryptochlorogenic acid
11	Bisabolol oxide B	36	Homovanillic acid
12	Azulene	37	Caffeic acid
13	4-Hydroxycoumarine	38	4-p-Coumaroylqunic acid
14	6-Hydroxycoumarine	39	Vanillic acid
15	7-Hydroxycoumarine	40	p-Coumaric acid
16	Luteolin	41	Ferulic acid
17	Patuletin	42	Rutin
18	Herniarin	43	Hesperetin
19	Apigenine-7-O-glucoside	44	Kaempferol-3-O-rutinoside
20	Apigenin-8-C-glucoside	45	Kaempferol-3-O-glucoside
21	alpha-Bisabolol, acetate	46	Populnetin
22	Gallic acid	47	Quercetin
23	Vanillic acid 4-β-D-glucoside	48	Naringenin
24	Syringic acid	49	Apigenin
25	Caffeic acid hexoside	50	Rosmarinic acid

**Table 2:** List of polyphenols, terpenes, sesquiterpenes, fatty acids, and flavonoids present in the UD aqueous extract as detected via LCMS/MS. CE stands for collision energy.

#	Compound	Ionization	Exact Mass	*m/z*	Signal Intensity	Ions	CE
1	3-Epinobilin	pos ESI	346.178	347.186	e^2^	247.133, 83.042	20
2	Bisabolol oxide B	pos ESI	238.193	239.200	e^3^	221.192, 81.071	20
3	7-Hydroxycoumarine	pos ESI	162.032	163.039	e^2^	119.049, 145.029	40
4	Luteolin	pos ESI	286.048	287.056	e^2^	153.019, 109.029, 213.055, 269.045	40
5	Patuletin	pos ESI	332.053	333.061	e^4^	109.029, 137.024, 183.029, 315.051 (10 EV)	30
6	Apigenin-8C-glucoside	pos ESI	432.106	433.113	e^2^	415.101, 397.124, 367.103 (10 EV)	5
7	Gallic acid	pos ESI	170.022	171.029	e^3^	153.019, 125.023	20
8	Vanillic acid-4-β-D-glucoside	pos ESI	330.290	331.295	e^2^	169.003	20
9	p-Hydroxybenzoic acid	pos ESI	138.032	139.039	e^4^	121.029, 95.050	20
10	Chlorogenic acid	pos ESI	354.095	355.103	e^3^	163.039, 337.092, 193. 071, 175.061	20
11	m-Hydroxybenzoic acid	pos ESI	138.032	139.039	e^4^	93.034	20
12	Homovanillic acid	pos ESI	182.058	183.066	e^3^	137.060, 165.055	20
13	Caffeic acid	pos ESI	180.042	181.044	e^4^	135.045, 163.039	15
14	4-O-beta-D-Glucosyl-4-coumaric acid	pos ESI	164.047	165.055	e^2^	91.054, 147.045, 119.049 (30 EV)	30
15	Rutin	pos ESI	610.153	611.161	e^1^	303.050	40
16	Kaempferol-3-O-rutinoside	neg ESI	594.158	593.151	e^3^	285.012, 257.121, 284.206	35
17	Kaempferol-3-O-glucoside	neg ESI	448.101	447.093	e^2^	284.024, 255.029, 227.034	35
18	Rosmarinic acid	neg ESI	360.085	359.077	e^1^	161.024, 359.077, 197.045, 135.071	40
19	Populnetin	neg ESI	286.048	285.042	e^2^	164.999, 255.029, 227.036, 117.035	20
20	Quercetin	neg ESI	302.043	301.037	e^2^	151.001, 178.996, 271.025	20
21	Apigenin	neg ESI	270.053	269.052	e^1^	117.038, 151.0080	30
22	Hesperetin	neg ESI	302.079	301.072	e^1^	136.017, 151.004, 164.012, 285.040	40
